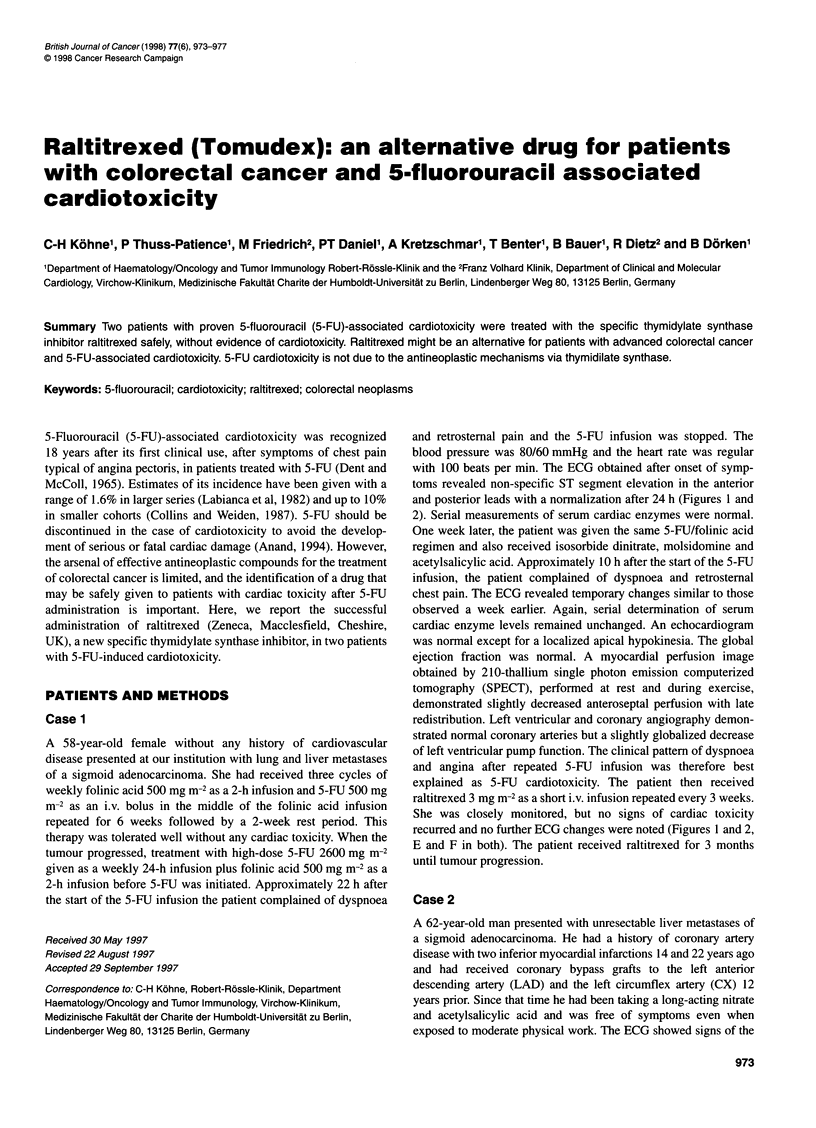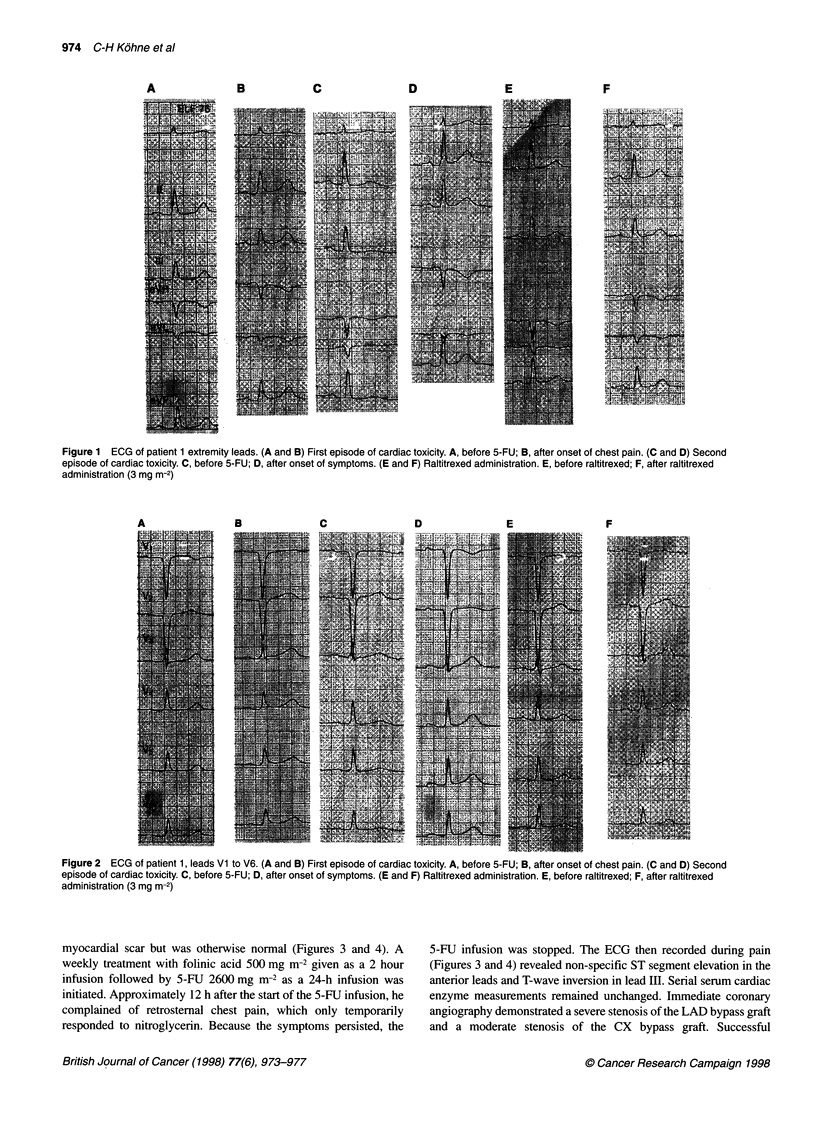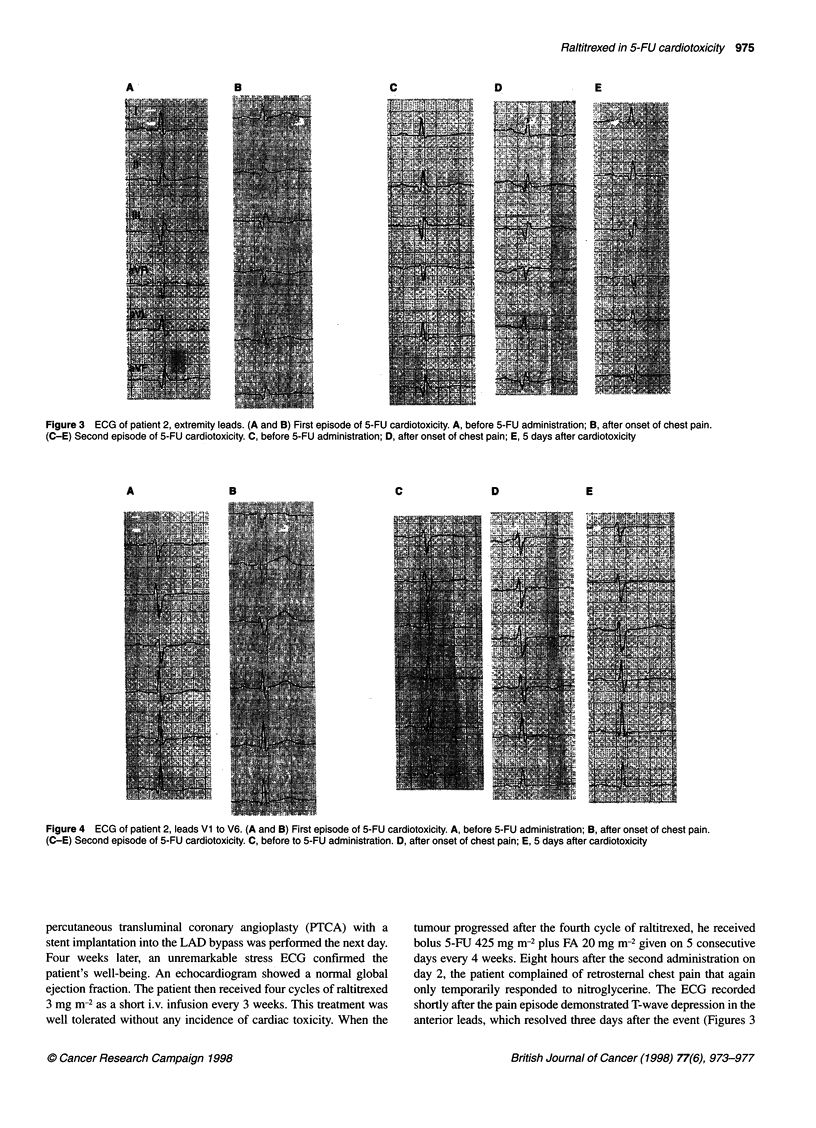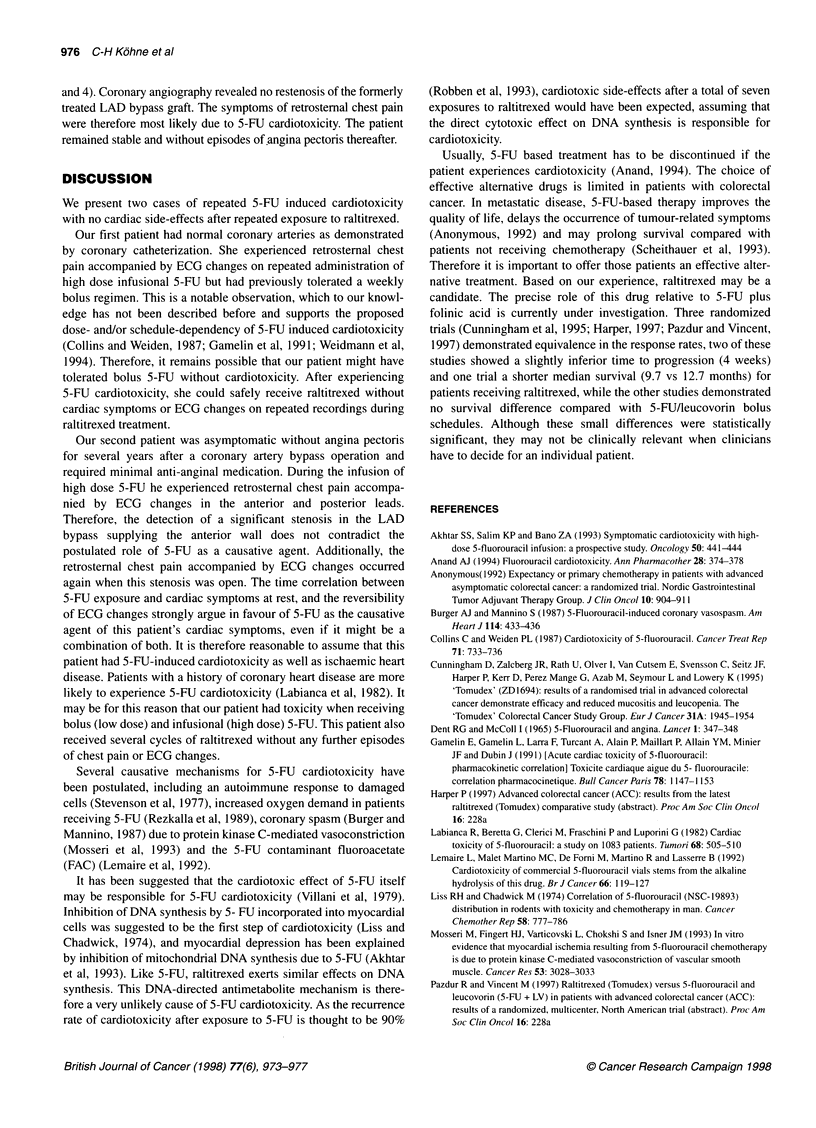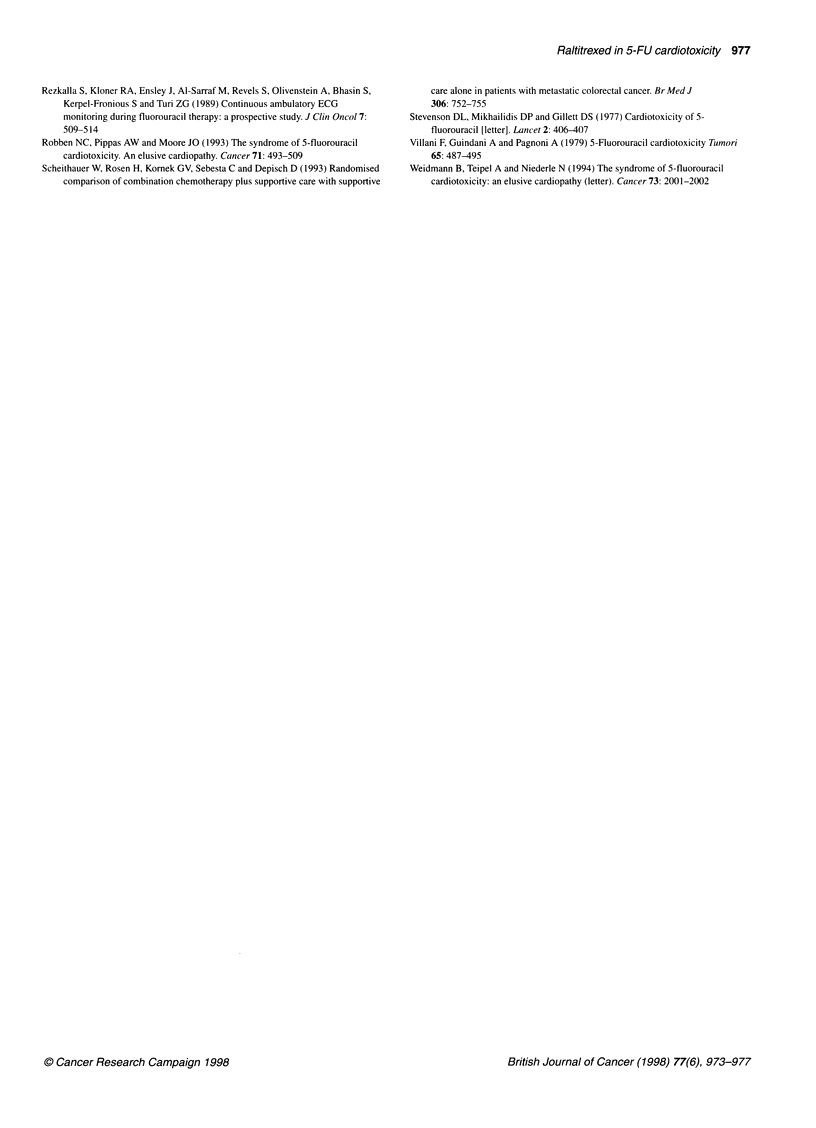# Raltitrexed (Tomudex): an alternative drug for patients with colorectal cancer and 5-fluorouracil associated cardiotoxicity.

**DOI:** 10.1038/bjc.1998.160

**Published:** 1998-03

**Authors:** C. H. KÃ¶hne, P. Thuss-Patience, M. Friedrich, P. T. Daniel, A. Kretzschmar, T. Benter, B. Bauer, R. Dietz, B. DÃ¶rken

**Affiliations:** Department of Haematology/Oncology and Tumor Immunology Robert-RÃ¶ssle-Klinik, Virchow-Klinikum, Medizinische FakultÃ¤t Charite der Humboldt-UniversitÃ¤t zu Berlin, Germany.

## Abstract

**Images:**


British Journal of Cancer (1998) 77(6), 973–977
© 1998 Cancer Research Campaign

### Raltitrexed (Tomudex): an alternative drug for patients with colorectal cancer and 5-fluorouracil associated cardiotoxicity

**C-H Köhne[1], P Thuss-Patience[1], M Friedrich[2], PT Daniel[1], A Kretzschmar[1], T Benter[1], B Bauer[1], R Dietz[2] and B Dörken[1]**

[1]Department of Haematology/Oncology and Tumor Immunology Robert-Rössle-Klinik and the [2]Franz Volhard Klinik, Department of Clinical and Molecular Cardiology, Virchow-Klinikum, Medizinische Fakultät Charite der Humboldt-Universität zu Berlin, Lindenberger Weg 80, 13125 Berlin, Germany

**Summary** Two patients with proven 5-fluorouracil (5-FU)-associated cardiotoxicity were treated with the specific thymidylate synthase inhibitor raltitrexed safely, without evidence of cardiotoxicity. Raltitrexed might be an alternative for patients with advanced colorectal cancer and 5-FU-associated cardiotoxicity. 5-FU cardiotoxicity is not due to the antineoplastic mechanisms via thymidilate synthase.

**Keywords:** 5-fluorouracil; cardiotoxicity; raltitrexed; colorectal neoplasms

5-Fluorouracil (5-FU)-associated cardiotoxicity was recognized 18 years after its first clinical use, after symptoms of chest pain typical of angina pectoris, in patients treated with 5-FU (Dent and McColl, 1965). Estimates of its incidence have been given with a range of 1.6% in larger series (Labianca et al, 1982) and up to 10% in smaller cohorts (Collins and Weiden, 1987). 5-FU should be discontinued in the case of cardiotoxicity to avoid the development of serious or fatal cardiac damage (Anand, 1994). However, the arsenal of effective antineoplastic compounds for the treatment of colorectal cancer is limited, and the identification of a drug that may be safely given to patients with cardiac toxicity after 5-FU administration is important. Here, we report the successful administration of raltitrexed (Zeneca, Macclesfield, Cheshire, UK), a new specific thymidylate synthase inhibitor, in two patients with 5-FU-induced cardiotoxicity.

### PATIENTS AND METHODS

### Case 1

A 58-year-old female without any history of cardiovascular disease presented at our institution with lung and liver metastases of a sigmoid adenocarcinoma. She had received three cycles of weekly folinic acid 500 mg $m^{-2}$ as a 2-h infusion and 5-FU 500 mg $m^{-2}$ as an i.v. bolus in the middle of the folinic acid infusion repeated for 6 weeks followed by a 2-week rest period. This therapy was tolerated well without any cardiac toxicity. When the tumour progressed, treatment with high-dose 5-FU 2600 mg $m^{-2}$ given as a weekly 24-h infusion plus folinic acid 500 mg $m^{-2}$ as a 2-h infusion before 5-FU was initiated. Approximately 22 h after the start of the 5-FU infusion the patient complained of dyspnoea and retrosternal pain and the 5-FU infusion was stopped. The blood pressure was 80/60 mmHg and the heart rate was regular with 100 beats per min. The ECG obtained after onset of symptoms revealed non-specific ST segment elevation in the anterior and posterior leads with a normalization after 24 h (Figures 1 and 2). Serial measurements of serum cardiac enzymes were normal. One week later, the patient was given the same 5-FU/folinic acid regimen and also received isosorbide dinitrate, molsidomine and acetylsalicylic acid. Approximately 10 h after the start of the 5-FU infusion, the patient complained of dyspnoea and retrosternal chest pain. The ECG revealed temporary changes similar to those observed a week earlier. Again, serial determination of serum cardiac enzyme levels remained unchanged. An echocardiogram was normal except for a localized apical hypokinesia. The global ejection fraction was normal. A myocardial perfusion image obtained by 210-thallium single photon emission computerized tomography (SPECT), performed at rest and during exercise, demonstrated slightly decreased anteroseptal perfusion with late redistribution. Left ventricular and coronary angiography demonstrated normal coronary arteries but a slightly globalized decrease of left ventricular pump function. The clinical pattern of dyspnoea and angina after repeated 5-FU infusion was therefore best explained as 5-FU cardiotoxicity. The patient then received raltitrexed 3 mg $m^{-2}$ as a short i.v. infusion repeated every 3 weeks. She was closely monitored, but no signs of cardiac toxicity recurred and no further ECG changes were noted (Figures 1 and 2, E and F in both). The patient received raltitrexed for 3 months until tumour progression.

### Case 2

A 62-year-old man presented with unresectable liver metastases of a sigmoid adenocarcinoma. He had a history of coronary artery disease with two inferior myocardial infarctions 14 and 22 years ago and had received coronary bypass grafts to the left anterior descending artery (LAD) and the left circumflex artery (CX) 12 years prior. Since that time he had been taking a long-acting nitrate and acetylsalicylic acid and was free of symptoms even when exposed to moderate physical work. The ECG showed signs of the

*Received 30 May 1997*
*Revised 22 August 1997*
*Accepted 29 September 1997*

*Correspondence to:* C-H Köhne, Robert-Rössle-Klinik, Department Haematology/Oncology and Tumor Immunology, Virchow-Klinikum, Medizinische Fakultät der Charite der Humboldt-Universität zu Berlin, Lindenberger Weg 80, 13125 Berlin, Germany

**Figure 1** ECG of patient 1 extremity leads. (**A** and **B**) First episode of cardiac toxicity. **A**, before 5-FU; **B**, after onset of chest pain. (**C** and **D**) Second episode of cardiac toxicity. **C**, before 5-FU; **D**, after onset of symptoms. (**E** and **F**) Raltitrexed administration. **E**, before raltitrexed; **F**, after raltitrexed administration (3 mg $m^{-2}$)

**Figure 2** ECG of patient 1, leads V1 to V6. (**A** and **B**) First episode of cardiac toxicity. **A**, before 5-FU; **B**, after onset of chest pain. (**C** and **D**) Second episode of cardiac toxicity. **C**, before 5-FU; **D**, after onset of symptoms. (**E** and **F**) Raltitrexed administration. **E**, before raltitrexed; **F**, after raltitrexed administration (3 mg $m^{-2}$)

myocardial scar but was otherwise normal (Figures 3 and 4). A weekly treatment with folinic acid 500 mg $m^{-2}$ given as a 2 hour infusion followed by 5-FU 2600 mg $m^{-2}$ as a 24-h infusion was initiated. Approximately 12 h after the start of the 5-FU infusion, he complained of retrosternal chest pain, which only temporarily responded to nitroglycerin. Because the symptoms persisted, the 5-FU infusion was stopped. The ECG then recorded during pain (Figures 3 and 4) revealed non-specific ST segment elevation in the anterior leads and T-wave inversion in lead III. Serial serum cardiac enzyme measurements remained unchanged. Immediate coronary angiography demonstrated a severe stenosis of the LAD bypass graft and a moderate stenosis of the CX bypass graft. Successful

© Cancer Research Campaign 1998

**Figure 3** ECG of patient 2, extremity leads. (**A** and **B**) First episode of 5-FU cardiotoxicity. **A**, before 5-FU administration; **B**, after onset of chest pain. (**C–E**) Second episode of 5-FU cardiotoxicity. **C**, before 5-FU administration; **D**, after onset of chest pain; **E**, 5 days after cardiotoxicity

**Figure 4** ECG of patient 2, leads V1 to V6. (**A** and **B**) First episode of 5-FU cardiotoxicity. **A**, before 5-FU administration; **B**, after onset of chest pain. (**C–E**) Second episode of 5-FU cardiotoxicity. **C**, before to 5-FU administration. **D**, after onset of chest pain; **E**, 5 days after cardiotoxicity percutaneous transluminal coronary angioplasty (PTCA) with a stent implantation into the LAD bypass was performed the next day. Four weeks later, an unremarkable stress ECG confirmed the patient's well-being. An echocardiogram showed a normal global ejection fraction. The patient then received four cycles of raltitrexed 3 mg $m^{-2}$ as a short i.v. infusion every 3 weeks. This treatment was well tolerated without any incidence of cardiac toxicity. When the tumour progressed after the fourth cycle of raltitrexed, he received bolus 5-FU 425 mg $m^{-2}$ plus FA 20 mg $m^{-2}$ given on 5 consecutive days every 4 weeks. Eight hours after the second administration on day 2, the patient complained of retrosternal chest pain that again only temporarily responded to nitroglycerine. The ECG recorded shortly after the pain episode demonstrated T-wave depression in the anterior leads, which resolved three days after the event (Figures 3

© Cancer Research Campaign 1998

and 4). Coronary angiography revealed no restenosis of the formerly treated LAD bypass graft. The symptoms of retrosternal chest pain were therefore most likely due to 5-FU cardiotoxicity. The patient remained stable and without episodes of angina pectoris thereafter.

### DISCUSSION

We present two cases of repeated 5-FU induced cardiotoxicity with no cardiac side-effects after repeated exposure to raltitrexed.

Our first patient had normal coronary arteries as demonstrated by coronary catheterization. She experienced retrosternal chest pain accompanied by ECG changes on repeated administration of high dose infusional 5-FU but had previously tolerated a weekly bolus regimen. This is a notable observation, which to our knowledge has not been described before and supports the proposed dose- and/or schedule-dependency of 5-FU induced cardiotoxicity (Collins and Weiden, 1987; Gamelin et al, 1991; Weidmann et al, 1994). Therefore, it remains possible that our patient might have tolerated bolus 5-FU without cardiotoxicity. After experiencing 5-FU cardiotoxicity, she could safely receive raltitrexed without cardiac symptoms or ECG changes on repeated recordings during raltitrexed treatment.

Our second patient was asymptomatic without angina pectoris for several years after a coronary artery bypass operation and required minimal anti-anginal medication. During the infusion of high dose 5-FU he experienced retrosternal chest pain accompanied by ECG changes in the anterior and posterior leads. Therefore, the detection of a significant stenosis in the LAD bypass supplying the anterior wall does not contradict the postulated role of 5-FU as a causative agent. Additionally, the retrosternal chest pain accompanied by ECG changes occurred again when this stenosis was open. The time correlation between 5-FU exposure and cardiac symptoms at rest, and the reversibility of ECG changes strongly argue in favour of 5-FU as the causative agent of this patient's cardiac symptoms, even if it might be a combination of both. It is therefore reasonable to assume that this patient had 5-FU-induced cardiotoxicity as well as ischaemic heart disease. Patients with a history of coronary heart disease are more likely to experience 5-FU cardiotoxicity (Labianca et al, 1982). It may be for this reason that our patient had toxicity when receiving bolus (low dose) and infusional (high dose) 5-FU. This patient also received several cycles of raltitrexed without any further episodes of chest pain or ECG changes.

Several causative mechanisms for 5-FU cardiotoxicity have been postulated, including an autoimmune response to damaged cells (Stevenson et al, 1977), increased oxygen demand in patients receiving 5-FU (Rezkalla et al, 1989), coronary spasm (Burger and Mannino, 1987) due to protein kinase C-mediated vasoconstriction (Mosseri et al, 1993) and the 5-FU contaminant fluoroacetate (FAC) (Lemaire et al, 1992).

It has been suggested that the cardiotoxic effect of 5-FU itself may be responsible for 5-FU cardiotoxicity (Villani et al, 1979). Inhibition of DNA synthesis by 5- FU incorporated into myocardial cells was suggested to be the first step of cardiotoxicity (Liss and Chadwick, 1974), and myocardial depression has been explained by inhibition of mitochondrial DNA synthesis due to 5-FU (Akhtar et al, 1993). Like 5-FU, raltitrexed exerts similar effects on DNA synthesis. This DNA-directed antimetabolite mechanism is therefore a very unlikely cause of 5-FU cardiotoxicity. As the recurrence rate of cardiotoxicity after exposure to 5-FU is thought to be 90% (Robben et al, 1993), cardiotoxic side-effects after a total of seven exposures to raltitrexed would have been expected, assuming that the direct cytotoxic effect on DNA synthesis is responsible for cardiotoxicity.

Usually, 5-FU based treatment has to be discontinued if the patient experiences cardiotoxicity (Anand, 1994). The choice of effective alternative drugs is limited in patients with colorectal cancer. In metastatic disease, 5-FU-based therapy improves the quality of life, delays the occurrence of tumour-related symptoms (Anonymous, 1992) and may prolong survival compared with patients not receiving chemotherapy (Scheithauer et al, 1993). Therefore it is important to offer those patients an effective alternative treatment. Based on our experience, raltitrexed may be a candidate. The precise role of this drug relative to 5-FU plus folinic acid is currently under investigation. Three randomized trials (Cunningham et al, 1995; Harper, 1997; Pazdur and Vincent, 1997) demonstrated equivalence in the response rates, two of these studies showed a slightly inferior time to progression (4 weeks) and one trial a shorter median survival (9.7 vs 12.7 months) for patients receiving raltitrexed, while the other studies demonstrated no survival difference compared with 5-FU/leucovorin bolus schedules. Although these small differences were statistically significant, they may not be clinically relevant when clinicians have to decide for an individual patient.

### REFERENCES

Akhtar SS, Salim KP and Bano ZA (1993) Symptomatic cardiotoxicity with high-dose 5-fluorouracil infusion: a prospective study. *Oncology* **50**: 441–444

Anand AJ (1994) Fluorouracil cardiotoxicity. *Ann Pharmacother* **28**: 374–378

Anonymous(1992) Expectancy or primary chemotherapy in patients with advanced asymptomatic colorectal cancer: a randomized trial. Nordic Gastrointestinal Tumor Adjuvant Therapy Group. *J Clin Oncol* **10**: 904–911

Burger AJ and Mannino S (1987) 5-Fluorouracil-induced coronary vasospasm. *Am Heart J* **114**: 433–436

Collins C and Weiden PL (1987) Cardiotoxicity of 5-fluorouracil. *Cancer Treat Rep* **71**: 733–736

Cunningham D, Zalcberg JR, Rath U, Olver I, Van Cutsem E, Svensson C, Seitz JF, Harper P, Kerr D, Perez Mange G, Azab M, Seymour L and Lowery K (1995) 'Tomudex' (ZD1694): results of a randomised trial in advanced colorectal cancer demonstrate efficacy and reduced mucositis and leucopenia. The 'Tomudex' Colorectal Cancer Study Group. *Eur J Cancer* **31A**: 1945–1954

Dent RG and McColl I (1965) 5-Fluorouracil and angina. *Lancet* **1**: 347–348

Gamelin E, Gamelin L, Larra F, Turcant A, Alain P, Maillart P, Allain YM, Minier JF and Dubin J (1991) [Acute cardiac toxicity of 5-fluorouracil: pharmacokinetic correlation] Toxicite cardiaque aigue du 5- fluorouracile: correlation pharmacocinetique. *Bull Cancer Paris* **78**: 1147–1153

Harper P (1997) Advanced colorectal cancer (ACC): results from the latest raltitrexed (Tomudex) comparative study (abstract). *Proc Am Soc Clin Oncol* **16**: 228a

Labianca R, Beretta G, Clerici M, Fraschini P and Luporini G (1982) Cardiac toxicity of 5-fluorouracil: a study on 1083 patients. *Tumori* **68**: 505–510

Lemaire L, Malet Martino MC, De Forni M, Martino R and Lasserre B (1992) Cardiotoxicity of commercial 5-fluorouracil vials stems from the alkaline hydrolysis of this drug. *Br J Cancer* **66**: 119–127

Liss RH and Chadwick M (1974) Correlation of 5-fluorouracil (NSC-19893) distribution in rodents with toxicity and chemotherapy in man. *Cancer Chemother Rep* **58**: 777–786

Mosseri M, Fingert HJ, Varticovski L, Chokshi S and Isner JM (1993) In vitro evidence that myocardial ischemia resulting from 5-fluorouracil chemotherapy is due to protein kinase C-mediated vasoconstriction of vascular smooth muscle. *Cancer Res* **53**: 3028–3033

Pazdur R and Vincent M (1997) Raltitrexed (Tomudex) versus 5-fluorouracil and leucovorin (5-FU + LV) in patients with advanced colorectal cancer (ACC): results of a randomized, multicenter, North American trial (abstract). *Proc Am Soc Clin Oncol* **16**: 228a

© Cancer Research Campaign 1998

Rezkalla S, Kloner RA, Ensley J, Al-Sarraf M, Revels S, Olivenstein A, Bhasin S, Kerpel-Fronious S and Turi ZG (1989) Continuous ambulatory ECG monitoring during fluorouracil therapy: a prospective study. *J Clin Oncol* **7**: 509–514

Robben NC, Pippas AW and Moore JO (1993) The syndrome of 5-fluorouracil cardiotoxicity. An elusive cardiopathy. *Cancer* **71**: 493–509

Scheithauer W, Rosen H, Kornek GV, Sebesta C and Depisch D (1993) Randomised comparison of combination chemotherapy plus supportive care with supportive care alone in patients with metastatic colorectal cancer. *Br Med J* **306**: 752–755

Stevenson DL, Mikhailidis DP and Gillett DS (1977) Cardiotoxicity of 5-fluorouracil [letter]. *Lancet* **2**: 406–407

Villani F, Guindani A and Pagnoni A (1979) 5-Fluorouracil cardiotoxicity *Tumori* **65**: 487–495

Weidmann B, Teipel A and Niederle N (1994) The syndrome of 5-fluorouracil cardiotoxicity: an elusive cardiopathy (letter). *Cancer* **73**: 2001–2002

*© Cancer Research Campaign 1998*